# A comparative study of high-dose-rate brachytherapy boost combined with external beam radiation therapy versus external beam radiation therapy alone for high-risk prostate cancer

**DOI:** 10.1093/jrr/rrab006

**Published:** 2021-04-05

**Authors:** Tomoya Oshikane, Motoki Kaidu, Eisuke Abe, Atsushi Ohta, Hirotake Saito, Toshimichi Nakano, Moe Honda, Satoshi Tanabe, Satoru Utsunomiya, Ryuta Sasamoto, Fumio Ishizaki, Takashi Kasahara, Tsutomu Nishiyama, Yoshihiko Tomita, Hidefumi Aoyama, Hiroyuki Ishikawa

**Affiliations:** Department of Radiology and Radiation Oncology, Niigata University Graduate School of Medical and Dental Sciences, 1-757 Asahimachi-dori, Chuo-ku, Niigata 951-8510, Japan; Department of Radiology and Radiation Oncology, Niigata University Graduate School of Medical and Dental Sciences, 1-757 Asahimachi-dori, Chuo-ku, Niigata 951-8510, Japan; Division of Radiation Oncology, Nagaoka Chuo General Hospital, 2041 Kawasaki-cho, Nagaoka 940-8653, Japan; Department of Radiology and Radiation Oncology, Niigata University Graduate School of Medical and Dental Sciences, 1-757 Asahimachi-dori, Chuo-ku, Niigata 951-8510, Japan; Division of Radiation Oncology, Niigata University Medical and Dental Hospital, 1-754 Asahimachi-dori, Chuo-ku, Niigata 951-8510, Japan; Department of Radiology and Radiation Oncology, Niigata University Graduate School of Medical and Dental Sciences, 1-757 Asahimachi-dori, Chuo-ku, Niigata 951-8510, Japan; Division of Radiation Oncology, Niigata University Medical and Dental Hospital, 1-754 Asahimachi-dori, Chuo-ku, Niigata 951-8510, Japan; Division of Radiation Oncology, Niigata University Medical and Dental Hospital, 1-754 Asahimachi-dori, Chuo-ku, Niigata 951-8510, Japan; Department of Radiological Technology, Niigata University Graduate School of Health Sciences, 2-746 Asahimachi-dori, Chuo-ku, Niigata 951-8518, Japan; Department of Radiological Technology, Niigata University Graduate School of Health Sciences, 2-746 Asahimachi-dori, Chuo-ku, Niigata 951-8518, Japan; Department of Urology, Niigata University Graduate School of Medical and Dental Sciences, 1-757 Asahimachi-dori, Chuo-ku, Niigata 951-8510, Japan; Department of Urology, Niigata University Graduate School of Medical and Dental Sciences, 1-757 Asahimachi-dori, Chuo-ku, Niigata 951-8510, Japan; Division of Urology, Uonuma Kikan Hospital, 4132 Urasa, Minami-Uonuma, Niigata 949-7302, Japan; Department of Urology, Niigata University Graduate School of Medical and Dental Sciences, 1-757 Asahimachi-dori, Chuo-ku, Niigata 951-8510, Japan; Department of Radiation Oncology, Faculty of Medicine, Hokkaido University, Kita 15, Nishi 7, Kita-ku, Sapporo, Hokkaido, Japan; Department of Radiology and Radiation Oncology, Niigata University Graduate School of Medical and Dental Sciences, 1-757 Asahimachi-dori, Chuo-ku, Niigata 951-8510, Japan

**Keywords:** high-dose-rate brachytherapy, intensity-modulated radiation therapy, prostate cancer, biochemical-free survival

## Abstract

We aimed to compare the outcomes of high-dose-rate brachytherapy (HDR-BT) boost and external beam radiation therapy (EBRT) alone for high-risk prostate cancer. This was a single-center, retrospective and observational study. Consecutive patients who underwent initial radical treatment by HDR-BT boost or EBRT alone from June 2009 to May 2016 at the Niigata University Medical and Dental Hospital, Japan were included. A total of 96 patients underwent HDR-BT boost, and 61 underwent EBRT alone. The prescription dose of HDR-BT boost was set to 18 Gy twice a day with EBRT 39 Gy/13 fractions. The dose for EBRT alone was mostly 70 Gy/28 fractions. The high-risk group received >6 months of prior androgen deprivation therapy. Overall survival, biochemical-free survival, local control and distant metastasis-free survival rates at 5 years were analyzed. The incidence of urological and gastrointestinal late adverse events of Grade 2 and above was also summarized. In the National Comprehensive Cancer Network (NCCN) high-risk calssification, HDR-BT boost had a significantly higher biochemical-free survival rate at 5 years (98.9% versus 90.7%, *P* = 0.04). Urethral strictures were more common in the HDR-BT boost group. We will continuously observe the progress of the study patients and determine the longer term results.

## INTRODUCTION

At present, there are multiple curative treatment methods for high-risk prostate cancer, but the choice depends on each facility, and there is no established standard. In previous studies, the biochemical-free survival rate of external beam radiation therapy (EBRT) combined with androgen deprivation therapy (ADT) at 5 years in high-risk patients has been reported to range from 70% to 88% [1–3]. On the other hand, in high-dose-rate brachytherapy (HDR-BT) boost combined with EBRT, the biochemical-free survival rate at 5 years has been reported to range from 78% to 89%, which shows a somewhat better treatment outcome than that for EBRT alone [4–8]. Our facility introduced HDR-BT in 2009 and started curative treatment of prostate cancer combined with three-dimensional conformal radiotherapy (3D-CRT) [9–12]. Subsequently, intensity-modulated radiotherapy (IMRT) was introduced in 2010 and started the curative treatment of prostate cancer. Thus, we have been treating prostate cancer with both modalities during the recent era. The study aim was to retrospectively compare the efficacy and safety results of HDR-BT boost and EBRT alone for high-risk prostate cancer at our institution.

## MATERIALS AND METHODS

### Background and target

Consecutive patients with high-risk prostate cancer, as determined according to National Comprehensive Cancer Network (NCCN) guidelines, who underwent initial radical treatment by HDR-BT boost or EBRT alone at the Niigata University Medical and Dental Hospital, Japan from June 2009 to May 2016 were included in this study. Patients at very high risk of prostate cancer were not included. Almost all patients were examined by magnetic resonance imaging (MRI) before all treatments, including ADT, and staging and lesion distribution data were recorded. A total of 96 patients underwent HDR-BT boost, and 61 underwent EBRT alone (Table 1).

**Table 1:** Patient characteristics of high-dose-rate brachytherapy (HDR-BT) boost group and external beam radiation therapy (EBRT) alone group. When comparing the distribution of T-stages between groups, there were more T3a cases in the HDR-BT boost group

Patient characteristics							
			**HDR-BT boost** (*n* = 96)		**EBRT alone** (*n* = 61)		
Parameter			Mean	(range)	Mean	(range)	*P* value
Age at RT (years)			68.5	47–79	70.0	60–84	0.01
Follow-up period (years)			5.04	1.27–8.97	3.91	0.67–6.52	<0.01
Initial PSA (ng ml^–1^)			16.4	3.0–138.2	18.4	5.5–135.9	0.56
					
T stage							
	T1c		7	7.3%	9	14.5%	0.01 (percentage with T3a)
	T2a		19	19.8%	18	29.5%	
	T2b		10	10.4%	8	13.1%	
	T2c		16	16.7%	11	18.0%	
	T3a		44	45.8%	15	24.6%	
ADT							
	No		18	18.7%	22	36.0%	<0.01
	pre + after RT		74	77.1%	31	50.1%	
	pre RT only		4	4.2%	8	13.1%	
Dose prescription		EQD2 (Gy)					
	39 Gy/13 fr + 18 Gy/2 fr	104	96	100.0%			
	70 Gy/28 fr	80			45	73.7%	
	70 Gy/35 fr	70			1	1.6%	
	67.5 Gy/27 fr	77			1	1.6%	
	65Gy/26fr	74			1	1.6%	
	62 Gy/20 fr	82			13	21.3%	

PSA, prostate-specific antigen; ADT, androgen deprivation therapy.

### Method of radiation therapy

All treatment plans were created by using 16-slice computed tomography (CT) (LightSpeed RT; General Electric Medical Systems, Waukesha, WI, USA), imaged at a field of view of 40 cm, a matrix of 512 × 512 and a slice thickness of 2.5 mm. Eclipse ver. 8.9.17 (Varian Medical Systems, Palo Alto, CA, USA) was used as the treatment planning device for external irradiation, the calculation algorithm was AAA ver. 8.9.17 and the grid size was 2.5 mm. No fiducial markers were used on the study subjects.

A Novalis TX (Varian) 6 MV or Clinac iX (Varian) 10 MV was used as a linear accelerator for EBRT performed prior to HDR-BT. The dose division of EBRT was fixed to 39 Gy/13 fractions [five times a week for 2.5 weeks, equivalent dose in 2 Gy fractions (EQD2) of 50 Gy when α/β = 1.5]. An isocenter prescription was adopted, and the planning target volume (PTV) included a margin of 8 mm from the prostate except for reduction to 6 mm on the dorsal side and inclusion of one-third of the seminal vesicles. The field adopted a combination of right and left opposite gates and pendulum irradiation. The schedule was set so that irradiation would be completed in principle 1 week before HDR-BT.

**Fig. 1.**
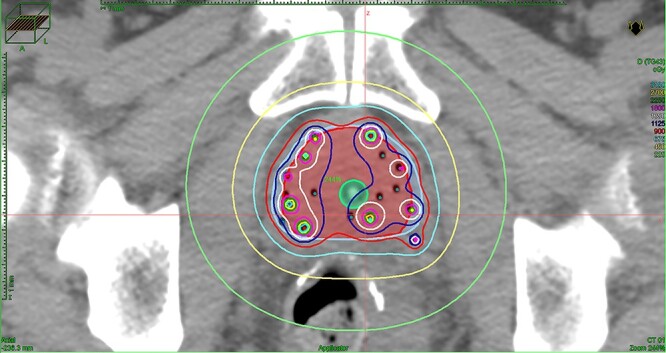
Dose distribution for a tumor in the right peripheral zone at the time of treatment planning. The central green structure represents the urethra, the outer red structure represents the prostate and the light purple structure represents the planning target volume.

The prescription dose of HDR-BT was set to 18 Gy twice a day (EQD2 of 54 Gy). Combined with EBRT and HDR-BT, the dose was equal to an EQD2 of 104 Gy. The interval time of irradiation was always set to >6 h. A urologist inserted ~18 plastic needle applicators by using the template from the patient’s perineum. As much as possible, the applicator was inserted in the area where the lesion was noted on the MRI. Plain CT was performed for each irradiation of HDR-BT [13], and the Oncentra Master Plan (Nucletron) was used as the treatment planning device. Each treatment plan was developed independently for twice-daily irradiation. The clinical target volume (CTV) in HDR-BT was defined in principle as the prostate and seminal vesicle base. The CTV and PTV were the same. The dose constraint was a PTV V100% ≥90%, a rectal V75% <1 ml and a urethral V125% <1 ml. For the bladder, dose restrictions were not placed, but we tried to reduce the prescription dose. As the prescription dose of 9 Gy to the PTV surface was administered, the dose distribution diagram was optimized manually for all patients while being mindful not to decrease the dose to the lesion recognized by MRI. Figure 1 shows an example of the dose distribution for a tumor in the right peripheral zone.

The EBRT alone was performed by using 6 MV X-rays of seven fixed fields (0°, 55°, 105°, 155°, 205°, 255° and 305°) on the Novalis TX with the IMRT technique. In treatment planning, contouring was performed with reference to the MRI. The CTV included the prostate plus about one-third of the proximal seminal vesicle base in T1 to T3a and the prostate plus the whole seminal vesicle in T3b. Dose splitting was basically 70 Gy/28 fractions (EQD2 of 80 Gy), but some variations were used early in the study period. The dose constraint of EBRT alone was set to D50% = 100% (99–101% allowed) with respect to the PTV, D95% >95%, maximum dose ≤107% and V90% ≥98%. Registration by tissue collation for all cases using cone beam CT (CBCT) was adopted. The patient was instructed not to urinate for 1–2 h prior to treatment. When feces and gas accumulation were observed during CBCT, we tried discharging as much as possible to prevent the lowering of positional accuracy because of rectal volume.

### ADT and follow-up

In our facility, for both treatment methods of HDR-BT boost and EBRT alone, ADT was prescribed for ≥6 months before radiotherapy and continued in principle for 3 years after radiotherapy for high-risk patients. Follow-up observations of physical findings and prostate-specific antigen (PSA) measurements were continued at intervals of once every 3 months for 2 years after radiotherapy and then every 6 months.

### Definition of recurrence

The follow-up period (up to March 2019) was calculated with the HDR-BT treatment day or EBRT end date as the start date. Biochemical recurrence was taken as the Phoenix definition (recurrence date with an increase of >2.0 ng ml^–1^ from the PSA nadir [14]) or the start or resumption of some medication therapy. Distant metastasis recurrence was defined as a lesion observed by any imaging findings. Prostate local recurrence was defined as a definitive distant/lymph node metastasis not confirmed in the imaging findings when PSA recurrence was confirmed. Prostate re-biopsy or prostate-specific membrane antigen positron emission tomography/computed tomography (PSMA-PET/CT) was not performed for cases of distant and local recurrence.

### Ethical approval

This research was approved by the ethics review committee for research at Niigata University, Japan [No. 2017-6218: Retrospective evaluation of treatment outcome of radiotherapy (HDR-BT and IMRT) for prostate cancer and quality of life].

### Statistical analysis

A significant difference test of the survival curve was performed by using the log-rank test for the two groups: HDR-BT boost and EBRT alone. For statistical analysis, EZR v1.51 [15] was used, and the significance level was set to 5%. The overall survival rate, biochemical-free survival rate, local control rate and distant metastasis-free survival rate were calculated using the Kaplan–Meier method.

Adverse events were evaluated by using CTCAE v4.0 and the electronic medical records. The most severe grades that occurred during the period were tabulated. Statistical analysis using the χ^2^ test was performed to determine if there was a significant difference in the proportions of Grade 3 or higher patients in each group.

## RESULTS

The median follow-up periods were 5.04 years and 3.91 years for HDR-BT boost and EBRT alone, respectively. With respect to T-stage, high-risk cases tended to be more common in the HDR-BT boost group, with 44 (45.8%) cases of T3a in the HDR-BT boost group and 15 (24.6%) cases in the EBRT alone group. Of the 96 patients who underwent HDR-BT boost, 78 (81.3%) used ADT. Among 61 patients who underwent EBRT alone, 39 (63.9%) used ADT. Significantly more patients in the HDR-BT boost group combined their treatment with ADT (*P* < 0.01) (Table 1).

**Fig. 2.**
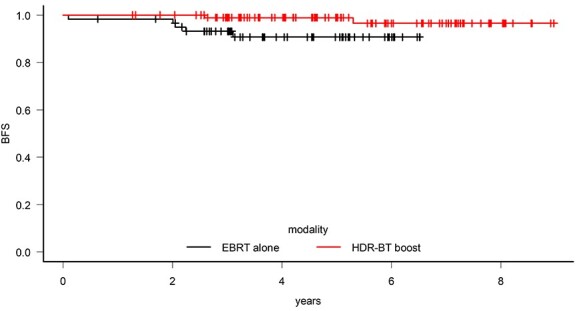
In high-risk prostate cancer patients, the addition of high-dose-rate brachytherapy (HDR-BT) boost significantly improved the biochemical-free survival (BFS) at 5 years compared with patients who received external beam radiation therapy (EBRT) alone.

In the HDR-BT boost group, two patients were biochemically recurrent, and both (100.0%) experienced local recurrence. Among the EBRT alone group, 5 patients were biochemically recurrent, including 2 (40.0%) patients with distant and lymph node recurrence and 3 (60.0%) patients with local recurrence. The mean time to biochemical recurrence was 3.95 years for HDR-BT boost and 1.90 years for EBRT alone. The mean time to local recurrence was 3.95 years for HDR-BT boost and 2.39 years for EBRT alone. The overall survival rate, biochemical-free survival rate, local control rate and distant metastasis-free survival rate at 5 years for HDR-BT boost/EBRT were 93.7%/90.6% (*P* = 0.88), 98.9%/90.7% (*P* = 0.04), 98.9%/94.0% (*P* = 0.22) and 100%/96.5% (*P* = 0.07), respectively (Table 2). The HDR-BT boost group had a significantly higher biochemical-free survival rate at 5 years (Fig. 2).

**Table 2:** The high-dose-rate brachytherapy (HDR-BT) boost group had a significantly higher biochemical-free survival (BFS) rate at 5 years. With respect to overall survival rate, local control rate and distant metastasis-free survival at 5 years, the differences were not significant

	HDR-BT boost (*n* = 96)		EBRT alone (*n* = 61)		*P* value
	**%**	**95% CI**	**%**	**95% CI**	
OS	93.7	85.2–97.4	90.6	72.0–97.1	0.88
BFS	98.9	92.3–99.8	90.7	78.9–96.1	0.04
LC	98.9	92.3–99.8	94.0	82.1–98.1	0.22
DMFS	100	N/A–N/A	96.5	86.8–99.1	0.07

OS, overall survival;

LC, local control;

DMFS, distant metastasis-free survival.

**Fig. 3.**
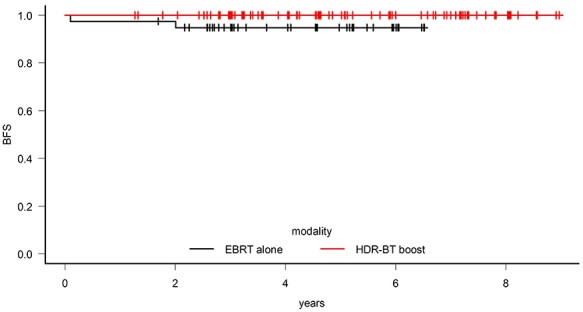
In high-risk prostate cancer patients who underwent androgen deprivation therapy (ADT), high-dose-rate brachytherapy (HDR-BT) boost significantly improved the biochemical-free survival (BFS) at 5 years compared with patients who received external beam radiation therapy (EBRT) alone.

Univariate analysis of the biochemical-free survival rate was performed on the subgroups of high-risk patients with and without ADT. When only the cases where treatments combined with ADT were examined, the biochemical-free survival rate at 5 years for the 78 HDR-BT boost patients and the 39 EBRT patients were 100% and 94.8%, respectively (*P* = 0.04). When analyzing between groups with ADT, HDR-BT boost had a significantly higher biochemical-free survival rate at 5 years (Fig. 3). When only cases that did not use ADT were examined, no significant differences in biochemical-free survival rate at 5 years were observed.

In late adverse events (AEs) of the urinary tract system, 36 Grade 2 (37.5%) and 10 Grade 3 (10.4%) AEs were observed in HDR-BT boost, and 10 Grade 2 (16.4%) and 1 Grade 3 (1.6%) AEs were observed in EBRT alone. In the late AEs of the digestive system, 10 Grade 2 (10.4%) and 1 Grade 3 (1.0%) AEs were observed in HDR-BT boost, and 11 Grade 2 (18.0%) and 2 Grade 3 (3.3%) AEs were observed in EBRT alone (Table 3). Late AEs of Grade 4 or higher were not observed in either group. When the ratio difference was examined for the incidence of AEs, the proportion of urinary late AEs with Grade 2 or more was significantly higher (*P* < 0.01) in HDR-BT boost and the proportion of late AEs in the digestive system was not significantly different (*P* = 0.113) between groups.

**Table 3:** Late adverse events (AE) [genitourinary (GU) and gastrointestinal GI)]. The proportion of urinary late AEs with Grade 2 or more was significantly higher (*P* < 0.01) in high-dose-rate brachytherapy (HDR-BT) boost and the proportion of late AEs in the digestive system was not significantly different (*P* = 0.113) between groups

		HDR-BT boost (*n* = 96)	EBRT alone (*n* = 61)
GU	Not observed	10 (10.4%)	19 (31.1%)
	Grade 1	401 (41.7%)	31 (50.8%)
	Grade 2	36 (37.5%)	10 (16.4%)
	Grade 3	10 (10.4%)	1 (1.6%)
	Grade 4, 5	0 (0%)	0 (0%)
GI	Not observed	42 (43.8%)	36 (59.0%)
	Grade 1	43 (44.8%)	12 (19.7%)
	Grade 2	10 (10.4%)	11 (18.0%)
	Grade 3	1 (1.0%)	2 (3.3%)
	Grade 4, 5	0 (0%)	0 (0%)

## DISCUSSION

In this study, we retrospectively compared the efficacy and safety of HDR-BT boost and EBRT alone for high-risk prostate cancer at our institution. When comparing the distribution of T-stages between groups, there were more T3a cases in the HDR-BT boost group. Despite this apparent disadvantage, patients in the HDR-BT group had better treatment outcomes. Research at the Memorial Sloan Kettering Cancer Center showed that HDR-BT combined with IMRT was superior to ultra-high-dose IMRT for a total of 630 patients, especially for NCCN intermediate-risk patients [16]. They reported no significant differences in high-risk patients, but the number of high-risk HDR-BT patients at that time was limited to 24. We included 96 NCCN high-risk HDR-BT patients in our study and observed a significantly higher biochemical-free survival rate in the HDR-BT boost group compared with the EBRT alone group.

Dose increase is considered effective for local control of prostate cancer [17], and the α/β of prostate cancer is assumed to be ~1.5 [18]. HDR-BT, which can immediately administer a high dose to a lesion, is claimed to be advantageous for local control from the viewpoint of radiation biology. In our facility, the EQD2 (α/β = 1.5) was 80 Gy for a dose division of 70 Gy/28 fractions for EBRT alone and 104 Gy for HDR-BT boost. Since the dose gradient of HDR-BT is steep compared with EBRT, it can irradiate a large dose to the prostate while reducing the rectal dose relatively easily. Another advantage of HDR-BT is that it is not necessary to consider the influence of organ movement in the body during treatment or error due to set-up. It is known that the prostate has some mobility and is deviated because of peristaltic movement of the rectum, the influence of gas and bladder volume [19–22]. Against this behavior, it is easy for HDR-BT to cover the prostate because of the characteristic that an applicator is inserted in the tissue. Therefore, CTV and PTV can be set equal. The ratio of the treatment intensity of EBRT and HDR-BT boost at our institution has been 50 Gy:54 Gy in terms of EQD2. HDR-BT (18 Gy/2 fractions/day) that can provide large doses at one time has high therapeutic strength. For these reasons, HDR-BT is theoretically an irradiation method that can provide high tumor control in prostate cancer [4–8].

We also found that the outcomes at our institution were better than those previously reported for high-risk prostate cancer [4–8, 23]. In previous reports, the biochemical-free survival rate for high-risk patients was 70–88% for EBRT combined with ADT, and 78–89% for HDR-BT boost. In contrast, we found a biochemical-free survival rate for high-risk patients of 90.7% for EBRT alone and 98.9% for HDR-BT boost. We would like to discuss the causes of these results. For HDR-BT, to the best of our ability, we inserted the applicator in the lesion that was recognized by the MRI. This approach ensured that the dose in the lesion would not be reduced, as the dose around the needle is inevitably higher. Higher doses to the lesion may have resulted in better control. In EBRT, we have devised certain measures, such as urinalysis/defecation instructions, MRI references at treatment planning and organizational verification of all cases using CBCT [20]. These approaches to guaranteeing the irradiation accuracy of EBRT may have led to better results for high-risk patients.

For late AEs, the incidence of urinary system (Grade 3) AEs in HDR-BT boost was 10.0% and significantly higher than the 3.3% found in EBRT alone. Among Grade 3 patients, all 10 in the HDR-BT group underwent internal urethrotomy due to urethral stricture. Frequent urinary catheterization is required for HDR-BT, which may explain the high rate of urethral stricture. The actual site of urethral stricture was often in the bulbar urethra, which did not receive a high dose. We took care that high doses of ≥125% of the prescribed dose did not enter the circumference of the urethra.

Regarding gastrointestinal AEs of Grade 3 and above, rectal bleeding, which was a concern, was not observed in either group. Recently we have started using spacers, and this will lead to more rectal protection.

There were several study limitations that should be considered. First, this was a single-center retrospective study, so selection bias may have occurred. In our institution, there was no clear criterion for distribution of treatment methods, and HDR-BT boost was mainly performed for high-risk patients. For this reason, there were few high-risk EBRT alone patients. Patients in poor health were more likely to be selected for EBRT alone. Second, the median follow-up period of this study was insufficient. In high-risk patients, ADT is continued in principle for 3 years after irradiation, so there is a possibility that eventual recurrence may be observed in some patients. To investigate the effectiveness of the treatment strategies of EBRT + ADT or EBRT + BT + ADT, as recommended by the NCCN guidelines, we performed a subgroup analysis comparing the cases in each group that received ADT. The biochemical recurrence-free survival rate was significantly better in the HDR-BT group. These results suggest the superiority of HDR-BT in combination with ADT in high-risk cases. Kishan *et al*. retrospectively examined outcomes of EBRT and EBRT + HDR-BT in multicenter patients with Gleason scores of 9–10 points and a median follow-up period of 5.1 and 6.3 years, respectively, and found that external irradiation + brachytherapy was superior in terms of 5-year prostate cancer-specific mortality and distant metastatic survival [24]. They stated that when evaluating high-risk treatment results, long-term follow-up may not be necessary before evaluating outcomes. In our study, further elongation of the follow-up period may show additional differences between the two treatment groups. Finally, patients with distant metastasis shown on imaging were not included in the local recurrence statistics. These patients were classified as experiencing distant metastasis recurrence, so the prostate was not biopsied again. For this reason, local recurrence may have been present in the distant metastasis patients, which could have influenced the local control rate.

We would like to offer our suggestions for dealing with high-risk prostate cancer based on our data and experience. First, we believe that EBRT + HDR-BT boost + ADT is the best treatment method for high-risk prostate cancer. We were able to achieve significantly better performance in biochemical recurrence-free survival of 5 years compared with EBRT + ADT. However, as there was no difference in the overall survival at 5 years, it must be considered that HDR-BT is not actively promoted as a treatment choice for patients whose expected prognosis is <5 years due to age or co-existing disease. Next, we were able to obtain good treatment results in this study because we were careful and well prepared in all aspects from planning to the time of actual irradiation. In almost all cases, MRI was conducted at the time of initial examination to determine the exact stage, and again at the time of EBRT planning to use as a reference for contouring. CBCT is conducted every time external irradiation is performed, and the location and contents of the rectum are controlled by defecation and exhaust gas. In HDR-BT, more attention is paid to the location where the applicator is inserted, and the dose to the lesion is not lowered. Such careful consideration will improve the quality of radiation therapy. Finally, it is important to consider the patient’s priorities in choosing a treatment option in terms not of only the patient’s overall condition but also of toxicity. It is good to explain to the patient in advance that the incidence of urethral stenosis in HDR-BT is significantly higher than that in EBRT alone.

## CONCLUSION

In patients with NCCN high-risk prostate cancer, HDR-BT boost had a significantly higher biochemical-free survival rate at 5 years (98.9% versus 90.7%, *P* = 0.04). Urethral strictures were more common in the HDR-BT boost group. We will continuously observe the progress of the study patients to determine the long-term outcomes.
